# Cell-free DNA sequencing *sheds* additional insights on *BRCA*-altered metastatic castration-resistant prostate cancer

**DOI:** 10.1016/j.ebiom.2023.104756

**Published:** 2023-08-09

**Authors:** Soha Bazyar, Philip Sutera, Matthew P. Deek, Catherine H. Marshall, Phuoc T. Tran

**Affiliations:** a Department of Radiation Oncology, University of Maryland School of Medicine, Baltimore, MD, USA; b Department of Radiation Oncology and Molecular Radiation Sciences, Johns Hopkins University School of Medicine, Baltimore, MD, USA; c Department of Radiation Oncology, Rutgers Robert Wood Johnson Medical School, Rutgers University, New Brunswick, NJ, USA; d Department of Oncology, Johns Hopkins University School of Medicine, Baltimore, MD, USA

Cancer of the prostate is the most common cancer occurring in men in the US. Despite advances in diagnosis and management, more than 34,000 men continue to die of prostate cancer each year in the United States.^1^ Androgen deprivation therapy (ADT) has been the standard of care for initial management of advanced or metastatic prostate cancer since Huggins and Hodges first introduced the concept of androgen-dependence. However, progression to metastatic castration-resistant prostate cancer (mCRPC) inevitably occurs after prolonged ADT. Tremendous progress has been made in the treatment of mCRPC in the form of life prolonging approved therapies, among them, taxanes and androgen receptor pathway inhibitors (ARPIs).^2^ Importantly, genomic studies of mCRPC have identified potentially actionable recurrent genomic aberrations. Loss-of-function alterations in DNA damage response (DDR) genes have been observed in greater than 20–25% of cases, most commonly defects in *BRCA* genes and other homologous recombination-mediated DNA damage response (HR-DDR) genes.^3^ In 2020, the FDA authorized two PARP inhibitor (PARPi) agents, olaparib and rucaparib, for the treatment of individuals with mCRPC who harbor genetic abnormalities affecting HR-DDR, following positive oncological outcomes in randomized clinical trials.^4^^,^^5^ Thus, molecular selection for PARPi treatment is currently the standard of care for mCRPC but less is known about the role of these gene mutations in clinical outcomes following non-PARPi treatment regimens. Recent clinical studies have also revealed that PARPi in combination with ARPIs in the first line setting can improve outcomes compared to PARPi alone, particularly in *BRCA*-mutated patients, leading to FDA approval of this combination in the mCRPC first-line setting for HR-DDR molecular subsets.^6^ Thus, easily identifying BRCA-deficiency and other potential targetable co-occurring genomic alterations in mCRPC is now of great importance.

Fettke and colleagues in this issue of *eBioMedicine* provide some additional insights into the landscape of DDR defects in mCRPC analyzing liquid biopsy and germline samples from a total of 375 men from the US and Australia using the CLIA-certified PredicineCARE™ cell-free DNA (cfDNA) panel assay which targets sequencing of 152 cancer driver genes including 27 individual DDR genes.^7^ From the 34% of the total cohort that exhibited DDR alterations, consistent with other studies, the most common altered gene was *BRCA2* (17%). Also consistent with some previous reports in *BRCA2* mutated mCRPC, clinical outcomes including PSA response, PFS and OS were found to be inferior with ARPI treatments.^3^^,^^6^ The most interesting finding from this predominantly cfDNA retrospective study, was that further genomic analysis indicated that identification of any pathogenic anomaly rather than specific *BRCA2* zygosity status (mono- or bi-allelic alterations) in cfDNA was indicative of poor clinical outcomes. This is interesting because previous data have suggested that bi-allelic *BRCA* altered mCRPC patients, particularly those with homozygous or bi-allelic *BRCA2* deletion, exhibited the most clinical benefit from PARPi. In contradistinction, previous studies have demonstrated that PARPi responses are rarely observed in patients without bi-allelic inactivating alterations in HR-DRR genes.^4^^,^^8^^,^^9^ Whether this discrepancy is simply the difference between prognostic and predictive biomarker associations or some more interesting foundational biological difference remains to be determined. Also, more research clearly needs to be performed to understand the differences in the cfDNA findings from this study by Fettke et al. and previous studies that used predominantly tissue-based genomic approaches.

As previously described, exploring different PARPi combination strategies will likely escalate as an important therapeutic option given recent positive results with PARPi and ARPI clinical studies.^6^ Fettke and colleagues also add to the growing realization that *BRCA2* altered mCRPC have a specific genomic background of co-occurring oncogenic drivers gene mutations, many of which are potentially actionable. They demonstrated using cfDNA sequencing that *BRCA2* altered mCRPC harbored mutations at approximately twice the frequency in potentially targetable pathways such as the AR, PI3K/PTEN, Fibroblast Growth Factor Receptor 1 (FGFR1) and CDK4/6 cell cycle pathways.

Overall, these findings from Fettke et al. support continued efforts suggesting that cfDNA approaches can reliably detect HR-DDR gene alterations with clinical implications in mCRPC patients.^10^ Interesting, they found *BRCA2* alterations of at least one allele within plasma cfDNA is sufficient to identify patients with inferior clinical outcomes, and that a distinct genomic landscape is present in patients with *BRCA2* altered mCRPC, with a high prevalence of potentially actionable oncogenic driver co-targets. Thus, this nice work by Fettke and colleagues with cfDNA has *shed* additional light on these potentially actionable co-targets with PARPi in *BRCA* altered mCRPC patients. However, much work remains to prospectively evaluate and validate co-targeting strategies with PARPi and the optimal sequencing of these strategies to increase treatment opportunities in mCRPC.

## Contributors

Literature search: S.B. and P.T.T.; Data collection: S.B. and P.T.T.; Data interpretation: S.B. and P.T.T.; Writing: S.B., P.S., M.P.D, C.H.M. and P.T.T. All authors read and approve the manuscript.

## Declaration of interests

CHM declares consulting fees for Bayer, Dendreon, Obseva and Astellas. PTT declares institutional grants from RefleXion Medical, Bayer and Astellas, Patent #9114158–Compounds and Methods of Use in Ablative Radiotherapy licensed to Natsar Pharm, consulting fees from RefleXion Medical, Janssen, AstraZeneca, Noxopharm, Myovant and Lantheus. The other authors declare no competing interests.
